# The Importance of Accounting for Parameter Uncertainty in SF-6D Value Sets and Its Impact on Studies that Use the SF-6D to Measure Health Utility

**DOI:** 10.3390/ijerph17113949

**Published:** 2020-06-02

**Authors:** Samer A. Kharroubi, Yara Beyh, Esmail Abdul Fattah, Tracey Young

**Affiliations:** 1 Department of Nutrition and Food Sciences, Faculty of Agricultural and Food Sciences, American University of Beirut, P.O.BOX: 11-0236, Riad El Solh, Beirut 1107-2020, Lebanon; eha11@mail.aub.edu; 2 Nutrition and Health Sciences, Laney Graduate School, Emory University, Atlanta, GA 30322, USA; yara@sbeyh.com; 3 Health Economics and Decision Science, School of Health and Related Research, The University of Sheffield, Regent Court, 30 Regent Street, Sheffield S1 4DA, UK; t.a.young@sheffield.ac.uk

**Keywords:** parameter uncertainty, health utility, Bayesian methods, SF-6D

## Abstract

Background: The parameter uncertainty in the six-dimensional health state short form (SF-6D) value sets is commonly ignored. There are two sources of parameter uncertainty: uncertainty around the estimated regression coefficients and uncertainty around the model’s specification. This study explores these two sources of parameter uncertainty in the value sets using probabilistic sensitivity analysis (PSA) and a Bayesian approach. Methods: We used data from the original UK/SF-6D valuation study to evaluate the extent of parameter uncertainty in the value set. First, we re-estimated the Brazier model to replicate the published estimated coefficients. Second, we estimated standard errors around the predicted utility of each SF-6D state to assess the impact of parameter uncertainty on these estimated utilities. Third, we used Monte Carlo simulation technique to account for the uncertainty on these estimates. Finally, we used a Bayesian approach to quantifying parameter uncertainty in the value sets. The extent of parameter uncertainty in SF-6D value sets was assessed using data from the Hong Kong valuation study. Results: Including parameter uncertainty results in wider confidence/credible intervals and improved coverage probability using both approaches. Using PSA, the mean 95% confidence intervals widths for the mean utilities were 0.1394 (range: 0.0565–0.2239) and 0.0989 (0.0048–0.1252) with and without parameter uncertainty whilst, using the Bayesian approach, this was 0.1478 (0.053–0.1665). Upon evaluating the impact of parameter uncertainty on estimates of a population’s mean utility, the true standard error was underestimated by 79.1% (PSA) and 86.15% (Bayesian) when parameter uncertainty was ignored. Conclusions: Parameter uncertainty around the SF-6D value set has a large impact on the predicted utilities and estimated confidence intervals. This uncertainty should be accounted for when using SF-6D utilities in economic evaluations. Ignoring this additional information could impact misleadingly on policy decisions.

## 1. Introduction

The need to appropriately quantify the health benefits produced by competing healthcare strategies in terms of quality-adjusted life years (QALYs) has become an increasingly important consideration for decision makers tasked with allocating healthcare funding. In the National Health Service (NHS) for England and Wales, the National Institute for Health and Care Excellence (NICE) considers the use of QALYs as a key requirement for any health technology submission undergoing the Institute’s appraisal process [1]. The QALY is a composite outcome that combines mortality and morbidity effects into a single numeraire. The most recent NICE guide to the methods of technology appraisal stipulates that morbidity should be measured using a preference-based measure of health-related quality of life (HRQoL), and currently recommends using the EuroQol five-dimensional (EQ-5D-3L) [2].

Heath state values deriving the QALYs incorporated in cost-effectiveness analysis (CEA) are obtained from a large variety of preference-based measures, including the EQ-5D questionnaire three-level and five-level versions [2], health utilities index 2 (HUI2) and 3 [3,4], 15D [5,6], Assessment of Quality of Life [7], Quality of Well-Being scale (QWB) Q5 [8] and the six-dimensional health state short form (derived from a short-form 36 health survey) (SF-6D) [9]. 

Measures of HRQoL such as the EQ-5D-3L, SF-6D, and HUI2 consist of a series of statements asking respondents to value their own health, for example the SF-6D consists of six statements, with between four and six response choices, giving a total of 18,000 health states. It is not feasible to value all possible health states, hence only a selection of these are typically measured. These states will have been selected based on designs, such as orthogonality, to ensure that those selected are representative of the full set of possible health states; they are also selected so that utility preference weights for all states can be estimated using statistical models. 

The statistical models fitted to health states (N3 model for EQ-5D-3L [10], SF-6D [9] and HUI2 [11]) will consist of one or more parameter estimates (known as preference weights) and, for each estimate, there will be a measure of uncertainty—for example, a standard error. When the preference weights (point estimates of the model parameters) are applied to other datasets to estimate utility values, which are then used to estimate QALYs, this measure of uncertainty is typically ignored. Although there is no clear reason for not including this information in tariffs associated with preference-based instruments, it is possible that at the time that methods for devising tariffs were developed, the methodological and computational challenges were insurmountable. Thus, economic evaluations that derive cost per QALY estimates from these models do not present the full uncertainty around the utility estimate, as noted by Ara and Wailoo [12]. 

The two main sources of parameter uncertainties were brought to light by Gray et al. [13], who distinguished between parameter uncertainty around the coefficients and model uncertainty around the model’s specifications. In fact, they deduced that parameter uncertainty around the coefficients is of little importance in the estimated confidence intervals for incremental QALY, and thus it could be ignored. However, Pullenayegum et al. [14] concluded that model uncertainty around the model’s specifications constitutes a major source of uncertainty in value sets, hence it is cardinal to the analysis. 

Probability distributions are commonly used to reflect uncertainty in the mean parameter estimates used to populate a cost-effectiveness model, and their impact on the study results is typically explored by means of probabilistic sensitivity analysis (PSA). This approach assigns appropriate distributions to model parameters and use simulation methods to capture uncertainty, in a similar way to those used by Young and Thompson [15] to capture model parameter uncertainty in published prognostic models. However, this approach only captures the sampling uncertainty that arises as a result of the study population completing a health-related quality of life instrument. Sampling uncertainty is inherent in the values assigned to individual health states arising from direct valuation methods, yet this is not captured under the current practice of ascribing point estimates to health state values. Additionally, there is no estimate of variance in those health state values currently derived through interpolation methods based on the sample of directly valued states. Alternatively, Pullenayegum et al. [14] developed a Bayesian approach to quantifying parameter uncertainty in the value sets, which accounts for parameter uncertainty around the estimated regression coefficients, and illustrated its impact on studies that use the EQ-5D-3L to measure health utility.

The aim of this paper is to illustrate the extent of parameter uncertainty through applying PSA and the Bayesian methods to explore the two sources of uncertainties in value sets when an HRQoL measure is used to compute the population’s mean utility. First, we will demonstrate that the parameter uncertainty in the Brazier model [9] creates discrepancies between the predicted and the observed mean utilities and serves as a guide for the PSA to propagate parameter uncertainty. Then, parameter uncertainty, accounting for both uncertainty around the coefficient (estimated in the Brazier model) and uncertainty around the model’s specifications, will be quantified using a Bayesian approach, allowing us to estimate parameter uncertainty for the Brazier model and show its impact on studies measuring health utility using the SF-6D. For this reason, we will use the SF-6D value set from the UK valuation study specifically gathered from a sample of 4596 Hong Kong (HK) general population using regression coefficients and their intercorrelation, and standard errors.

## 2. Methods

### 2.1. The SF-6D

The SF-6D stands for the short-form six-dimensions value set, derived from the original short-form 36 (SF-36). It is composed of six health dimensions, including physical functioning, role limitation, social functioning, bodily pain, mental health and vitality, each having between four and six levels [9]. Defining a health state requires choosing a level from each dimension, hence creating 18,000 possible combinations. Since every possible health state is described by six digits, from 1 to 6, thus the perfect health state (full health) is indicated by the combination 111111, whereas the “pits” (worst health state) is indicated by 645655. 

### 2.2. The Valuation Survey and Data Set

#### 2.2.1. UK

The SF-6D UK value set was derived from a sample of 249 health states described through the SF-6D and then valued by a representative sample of the UK population (*n* = 836). The selection methods of respondents along with health states are discussed elsewhere [9]. Of the original 836 respondents, a total of 225 respondents had to be excluded for several reasons. Each of the total 611 included respondents valued six health states according to the McMaster ‘ping pong’ variant of the standard gamble (SG) technique, giving 3666 valuations. Of these, 148 missing values from 117 respondents were present, thereby resulting in a total of 3518 observed SG valuations across the 249 health states. Further details pertaining to the valuation of the 249 SF-6D UK health states can be found in [9].

#### 2.2.2. Hong Kong

The HK study comprised a sample of 197 health states (selected using the same approach as the UK procedures) which were valued using the same valuation procedures as those in the UK study [16]. Each respondent was asked to rank and value eight health states, and the interview procedure was modelled on the basis of that in the UK study. Out of the original 641 respondents, a total of 59 respondents were disqualified, leaving 582 respondents’ data for the analysis. Each of the 582 respondents made eight SG valuations, giving 4596 valuations. Of these, 60 missing health state values were present and so 4596 observed SG valuations across 197 health states were finally included in the analysis. A detailed description to the valuation of the 197 SF-6D HK health states has been reported elsewhere [16].

#### 2.2.3. Modelling

Generally, a model for health state valuations can be expressed as

(1) $$y_{ij}=f(x_{ij},\alpha_{j})+\varepsilon_{ij}$$

where, for *i* = 1, 2, …, $n_{j}$ and *j* = 1, 2, …, *m*, **x***_ij_* represents the *i*th health state valued by respondent *j* with the dependent variable *y_ij_* being the adjusted SG score. Two sets of zero-mean, independent terms of random effects are involved in this general model. First, $\varepsilon_{ij}$ is a random observation-related error term, while $\alpha_{j}$ is a respondent-*j*-related term accounting for the individual characteristics. 

Respondent j’s health state utility has been defined by Brazier et al. [9] by the following model

(2) $$f(x_{ij},\alpha_{j})=\mu +\theta^{\prime }I(x_{ij})+\alpha_{j}$$

where μ and θ are unknown parameters, and $I(x_{ij})$ is a vector of dummy explanatory variables. Considering a simple case with no interactions, $I(x_{ij})$ would be a vector of terms $I_{\delta \lambda }(x_{ij})$ for each level λ > 1 of dimension δ of the SF-6D. For instance, *I*_32_(**x**_*ij*_) is level λ = 2 (health limits social activities a little of the time) of dimension δ = 3 (social functioning). Moreover, for any given health state **x**_*ij*_, $I_{\delta \lambda }(x_{ij})$ is defined as:

$$I_{\delta \lambda }(x_{ij})=1\;\mathrm{if},\;\mathrm{for}\;\mathrm{state}\;x_{ij},\;\mathrm{dimension}\;\delta \;\mathrm{is}\;\mathrm{true}\;\mathrm{at}\;\mathrm{level}\;\lambda$$

$$I_{\delta \lambda }(x_{ij})=0\;\mathrm{if},\;\mathrm{for}\;\mathrm{state}\;x_{ij},\;\mathrm{dimension}\;\delta \;\mathrm{is}\;\mathrm{not}\;\mathrm{true}\;\mathrm{at}\;\mathrm{level}\;\lambda$$

Overall, the 25 defined terms have level λ = 1 as the baseline for each dimension. Hence, the health state utility value for state 111111 is represented by the intercept parameter μ, which, by adding it to the sum of the coefficients $\theta_{\delta \lambda }$ of the ‘on’ dummies, derives the value of any other state. For example, health state utility value for state 111215 is: $\mu +\theta_{42}+\theta_{65}$.

Broadly, interactions between the levels of different dimensions can be accounted for by including other dummy variables in $I(x_{ij})$. The term MOST, in the model selected by Brazier et al. [9], fulfilled this action by having a value of 1 should any dimension in the health state be at one of the most severe levels (Most severe is defined as levels 4 to 6 for physical functioning, levels 3 and 4 for role limitation, 4 and 5 for social functioning, mental health and vitality, and 5 and 6 for pain.), and 0 in other cases. 

The generalized least square (GLS) model and maximum likelihood can estimate this random effect model. Additionally, since $\alpha_{j}$ has a mean of zero, then the population health state utility for state **x** in this model is simply expressed as $\mu +\theta^{\prime }I(x)$.

However, the within- and between-respondent error terms are connected in those models, hence the Random Effect (RE) model, an improved specification, separates those error terms by acknowledging that the error may not be independent of the respondent, namely

$$u_{i}+e_{ij}$$

where *u_i_* is the respondent specific variation, assumed to be random across individual respondents, and *e_ij_* is an error term for the *j*th health state valuation of the *i*th individual, assumed to be random across observations. Additionally, this model assumes that the allocation of health states to respondents is random, i.e., cov(*u_i_, e_ij_*) = 0.

Here, the re-estimation of the Brazier model (Equation (2)) from the original data provided a covariance matrix and standard errors for each coefficient. This re-estimation was used to compute mean utility values and respective standard errors for each of the 249 health states of the valuation study. Despite the misspecification resulting from omitted variables or incorrect functional errors in the published Brazier model, the main aim of this paper is to examine the consequences of incorporating parameter uncertainty in the existing model rather than to propose a different model specification. 

#### 2.2.4. PSA Approach to Parameter Uncertainty in the Value sets 

Economic valuations calculate utilities for patients based on the point estimate of the utility for each health state, while ignoring the existing parameter uncertainty in the value sets. In this paper, the impact of parameter uncertainty is generally examined in the SF-6D UK valuations on health utility measurement, and particularly quantified in the valuations when the SF-6D is used to measure a population’s mean utility.

#### 2.2.5. HK Population’s Mean Utility Using Regression Coefficients Only

The mean utility and 95% confidence interval for the HK survey population became readily available through applying the derived UK model to the HK survey population. In other terms, the value of any of the HK states in the HK study were derived by summing the coefficients $\theta_{\delta \lambda }$ of the ‘on’ dummies, using only the regression coefficients obtained from the UK model.

#### 2.2.6. HK Population’s Mean Utility Using Regression Coefficients and Their Standard Errors

The uncertainty in the regression coefficients was accounted for using the Monte Carlo simulation techniques, which resulted in the generation of 10,000 sets of multivariate normally distributed regression coefficients. The means and standard errors of the 10,000 generated sets mimicked those of the sets derived from the RE model fitted to the UK data. The statistical computer package R was used to generate these results. After using all the 10,000 sets of regression coefficients to derive UK model scores, they were applied to the HK cohort in order to generate 10,000 sets for the HK survey population, for which the mean utilities, overall mean utilities and 95% confidence intervals are readily available.

#### 2.2.7. Bayesian Approach to Parameter Uncertainty in the Value Sets 

We then used a Bayesian approach similar to the approach used by Pullenayegum et al. [14] to quantify parameter uncertainty in the value sets, considering both parameter uncertainty around the regression coefficients and parameter uncertainty around the specification of the model. Bayesian analysis rests upon computing the posterior probability distribution for model parameters, thus making it easy to characterize parameter uncertainty. It is in this sense that Bayesian methods are getting very popular in economic evaluations.

For respondent *j*, the health state utility of state **x**_*ij*_ is

(3) $$f\left(x_{ij},\delta_{i}\right)=\mu +\theta^{\prime }I\left(x_{ij}\right)+\alpha_{j}+\delta_{i}$$

where $\delta_{i}$ acknowledges the possibility of model misspecification, which is equal to zero i.e., $\delta_{i}=0$ for all *i* if there is no model misspecification. We assigned independent normal distributions to the respondent residual and model misspecification terms

$$\delta_{i}\sim N\left(0,\sigma_{\delta }^{2}\right),\;\alpha_{j}\sim N\left(0,\sigma_{\alpha }^{2}\right)$$

where $\sigma_{\delta }^{2}$ and $\sigma_{\alpha }^{2}$ are further parameters to be estimated. 

The specialist software WinBUGS (version 1.4; MRC Biostatistics unit: Cambridge, UK) facilitated the fitting of the model using the Bayesian Markov Chain Monte Carlo (MCMC) simulation method [17,18]. The relevant code is available from the corresponding author. The prior distributions for all the regression coefficients were defined to be Normal (0, 10^6^); in other words, they were centered on 0 with a large non-informative variance. The latter are not defined in WinBUGS, but instead precisions are specified as τ = 1/σ^2^. Since τ is the inverse of a variance parameter (always an absolute value), an additional minimally informative prior was required, hence, the common choice of Gamma (0.001, 0.001).

$$\sigma_{\delta }^{2},\sigma_{\alpha }^{2}\sim \mathrm{InverseGamma}(0.001,0.001)$$

Initially, 10,000 iterations were run as a “burn in” for the MCMC sampler to reach convergence, which was assessed by Gelman and Rubin diagnostic [19]. The process involved starting two parallel chains from scattered starting values and monitoring the within-chain and between-chain variance until reaching convergence at 1. Afterwards, an additional 10,000 iterations were run for parameters estimation purposes. 

We next considered the impact of parameter uncertainty in the value sets on studies using the SF-6D to estimate mean health utility. In particular, we quantified the impact of parameter uncertainty in the valuations when the SF-6D is used to measure the mean utility of a population. To meet this objective, we used the UK valuation study to estimate utility values for the full set of health states and then applied this value set to SF-6D health states collected from a sample of 4596 HK general population.

The mean utility for the SF-6D HK population was computed by first using the valuation sample to estimate utility values for the full set of health states and then fitting the Pullenayegum model [14] to the HK data as follows

*Utility_j_ = f_i(j)_*

where *i(j)* is subject *j*’s health state and *j* = 1, ..., 4596

*i(j)~Multinomial(p)*

where *p* is a vector of probabilities where the *i*th element *p_i_* represents the probability of a randomly selected respondent having self-reported health state *i*, and prior information

$$p\sim Dirichlet\;(\alpha ),\;\alpha =\left(\alpha_{1},\alpha_{2},\ldots ,\alpha_{18000}\right)$$

with $\alpha_{1}$ = 1/18000 for *i* = 1, …, 18000. Therefore, the mean utility $\bar{f}$ for the SF-6D HK population is $\sum_{i=1}^{18000}p_{i}u_{i}$ with variance

(4) $$\mathrm{var}\left(\bar{f}\right)=E\left\{\mathrm{var}(\bar{f}|\mathrm{value}\;\mathrm{set})\right\}+\mathrm{var}\{E(\bar{f}|\mathrm{value}\;\mathrm{set})\}$$

As already mentioned in [14], the variance in the mean utility $\bar{f}$ for the SF-6D HK respondents is equal to the variance in the sample mean when ignoring parameter uncertainty in the value set plus the variance in the sample mean as the value set varies over its posterior distribution. 

## 3. Results 

### Parameter Uncertainty in the SF-6D UK Value Set 

**Figure 1:**
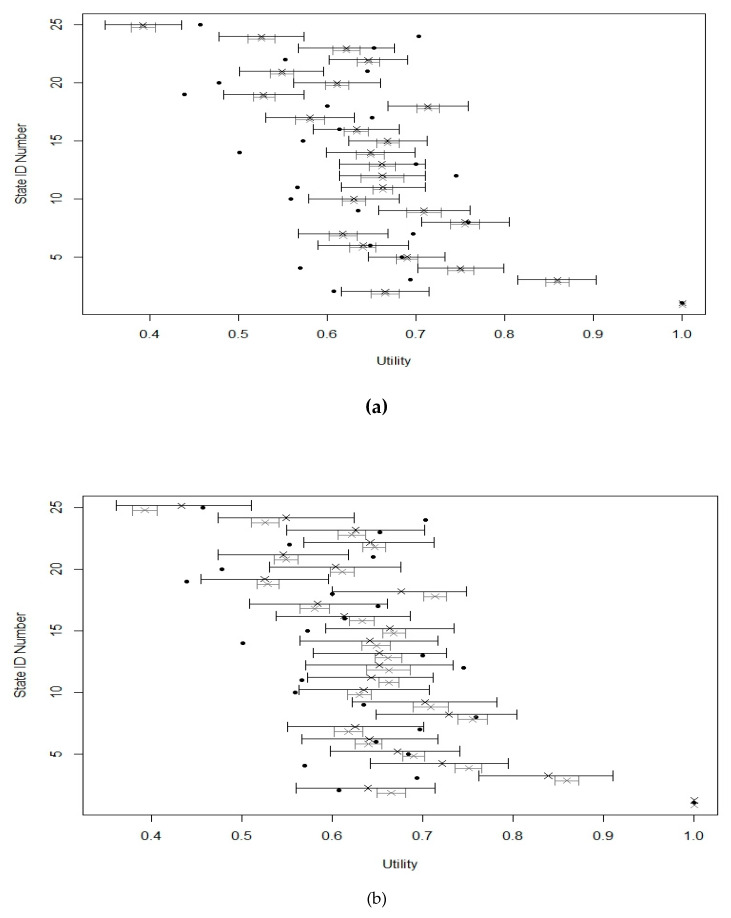
Estimates and 95% intervals for the 25 selected health states. The black circles give the observed mean utilities for each valued health state. The gray crosses and lines indicate the predicted means and 95% confidence intervals, ignoring parameter uncertainty. The black lines are the 95% confidence intervals from (**a**) the probabilistic sensitivity analysis (PSA) approach and (**b**) the Bayesian approach, respectively, that acknowledge uncertainty.

In total, 25 health states valued in the UK sample were systematically selected from the initial 249 health states. Table 1 shows the observed sample mean health state utility and the predicted mean and standard deviation for the population’s mean health state utility with and without parameter uncertainty for the 25 health states. As observed, the standard errors of the mean utilities for all health states when parameter uncertainty is tolerated using the PSA approach were larger than those ignoring parameter uncertainty. Therefore, the mean utilities of the 25 valued health states have slightly wider 95% confidence intervals than those without parameter uncertainty. This is reflected in Figure 1a, showing the estimates and 95% confidence intervals for the mean utilities of the 25 health states. Evidently, tolerating the possibility of parameter uncertainty improves coverage probability through wider confidence intervals.

The Supplementary Material shows the inference for the mean health state utility values of the 249 health states valued in the sample. Across all 249 states, the mean 95% confidence intervals widths are 0.0942 (range: 0.0480–0.1149) and 0.0285 (range: 0.002–0.0483) with and without parameter uncertainty, respectively, with a difference of 0.0657. Further, only 15% of the observed means fell within the 95% CI when ignoring parameter uncertainty, in comparison to 36% when parameter uncertainty is considered.

We now use a Bayesian approach to quantifying parameter uncertainty in the value sets, considering both parameter uncertainty around the regression coefficients and parameter uncertainty around the specification of the model. The final two columns of Table 1 present the posterior predicted mean and standard deviation for the population mean health state utility $f\left(x_{ij},\delta_{i}\right)$ of those 25 health states included in the valuation study. Acknowledging the possibility of model misspecification leads to a further increase in the standard errors of the mean unities for all health states, and therefore wider credible intervals and improved coverage probability are obtained. This can also be seen from Figure 1b, which shows the estimates and 95% confidence intervals for the mean utilities of the 25 selected health states. As can be seen, acknowledging the possibility of model uncertainty leads to wider confidence intervals and improved coverage probability. Finally, across all 249 states that were used in the study, the mean 95% credible intervals width is 0.1478 (range: 0.053–0.1665), and 54% of the observed means fell within the 95% credible intervals.

We finally quantify the impact of parameter uncertainty in the valuations when the SF-6D is used to estimate a population’s mean utility. This is achieved by using the UK valuation study to estimate utility values for the full set of health states and then applying this value set to SF-6D health states obtained from a sample of 4596 HK general population. Given the HK valuations and using the PSA approach, the mean health utility was found to be 0.576 with a standard error of0.001689. Ignoring parameter uncertainty in the value set, the standard error of the measurement sample’s mean utility was found to be 0.000353. This implies that the true standard error was underestimated by 79.1% when parameter uncertainty was not accounted for. This is also the case using the Bayesian approach, as the true standard error was underestimated by 86.15% when ignoring parameter uncertainty (see Table 2).

## 4. Discussion

In this paper, we have explored the two sources of uncertainty: (1) parameter uncertainty around the coefficients estimated in the Brazier et al. model and (2) model uncertainty around the specification of the model used to estimate the Brazier et al. model. We have used PSA and Bayesian methods to account for both types of uncertainty simultaneously in the value set when a measure of HRQoL (SF-6D here) is used to measure a population’s mean utility. We have shown, using both approaches, that parameter uncertainty around the value sets is substantial and should not be ignored in economic evaluations, and thus should be fully represented when reporting results. This finding is in line with previous work [13] which suggested that the impact of parameter uncertainty due to scoring was the largest for small valuation studies and that the uncertainty around the specification of the model was found to be a larger contributor to this parameter uncertainty [14].

We have illustrated how acknowledging the possibility of parameter uncertainty leads to wider confidence intervals and improved coverage probability using both approaches. Across all 249 states, the mean 95% credible intervals widths were 0.0942 (range: 0.0480–0.1149) and 0.0285 (range: 0.002–0.0483) with and without parameter uncertainty, respectively, using PSA. Whilst using the Bayesian approach, the mean 95% credible intervals widths was 0.1478 (range: 0.053–0.1665). Further, only 15% of the observed means fell within the 95% CI when ignoring parameter uncertainty, in comparison to 36% and 54% when parameter uncertainty is considered, using the PSA and Bayesian approaches, respectively. Finally, upon quantifying the impact of parameter uncertainty in the valuations when the SF-6D is used to estimate a population’s mean utility, the true standard error was underestimated by 79.1% and 86.15% when parameter uncertainty was not accounted for using the PSA and Bayesian approaches, respectively. 

We have shown that the Bayesian approach is superior to the frequentist PSA equivalent for our presented results as it led to wider credible intervals and improved coverage probability. Another potential advantage of the Bayesian model is its ability to produce probability distributions describing the uncertainty in the expected health state values—an increasingly important input to cost effectiveness analyses for health technology assessment. Although it is common that parameter standard error estimates give some clue about the uncertainty of estimates, the posterior distributions capture the full range of uncertainty inherent in these utility estimates. For example, they provide estimates of the uncertainty in the health state predictions from the model, which the frequentists cannot do. This leads to the conclusion that the Bayesian method is more flexible in characterizing inputs to regression models and more comprehensive in characterizing the uncertainty in the model outputs.

Given the centrality of either EQ-5D or SF-6D and the QALY in the international health policy context, an accurate estimation of health state utility values, along with an understanding of the uncertainty inherent in these values, becomes paramount for ensuring efficiency in the allocation of healthcare resources. We have illustrated how parameter uncertainty in the value set can be accounted for in both PSA and in Bayesian analysis. Currently, parameter uncertainty around the value sets tends to be ignored in economics evaluations. As a result, this gives committees on reimbursement a false level of confidence in the evidence and risks reimbursing interventions that are not cost-effective [14].

Following the development of revised tariffs in this research, it will be necessary to ensure that these can be applied in decision analysis modelling—that is, to explore the implications for decision uncertainty of using the newly devised method through a comparison of cost-effectiveness analyses conducted using a standard index value set compared with the revised set developed here. There is a scope for incorporating the novel methods within economic evaluations using patient-level data [20,21,22,23]. Such evaluations use individual patient health state values in estimating cost-effectiveness. It is anticipated that in these circumstances the substitution of a fixed utility estimate with the uncertain estimate will be methodologically straightforward. Further research is required to clarify this.

## 5. Conclusions

In conclusion, this article evaluates the importance of accounting for parameter uncertainty around the SF-6D value sets and its impact on studies that use the SF-6D to measure health utility. We have shown, using two different approaches: PSA and a Bayesian approach, that parameter uncertainty around the value sets is substantial and should not be ignored in economic evaluations, and thus should be fully represented when reporting results. Ignoring this additional information could impact misleadingly on policy decisions. 

## Figures and Tables

**Table 1:** Inference for a selection of health states.

			Brazier Model [9]		Probabilistic Sensitivity Analysis (PSA)		Bayesian	
Health State (HS) Number	HS	Observed	Predicted	Standard Deviation (SD)	Predicted	SD	Predicted	SD
1	111111	1.0000	1.0000	0.0000	1.0000	0.0000	1.0000	0.0000
2	112543	0.6070	0.6648	0.0252	0.6648	0.0253	0.6386	0.0393
3	122211	0.6927	0.8588	0.0227	0.8590	0.0224	0.8384	0.0377
4	133132	0.5691	0.7503	0.0247	0.7500	0.0246	0.7212	0.0391
5	144144	0.6842	0.6896	0.0221	0.6897	0.0219	0.6718	0.0364
6	212453	0.6483	0.6396	0.0259	0.6393	0.0261	0.6406	0.0381
7	221535	0.6960	0.6174	0.0255	0.6171	0.0254	0.6247	0.0387
8	232111	0.7589	0.7550	0.0251	0.7550	0.0247	0.7283	0.0395
9	244313	0.6343	0.7085	0.0265	0.7083	0.0263	0.7023	0.0406
10	315515	0.5587	0.6295	0.0259	0.6294	0.0258	0.6342	0.0370
11	323153	0.5656	0.6623	0.0241	0.6621	0.0240	0.6427	0.0357
12	331244	0.7450	0.6620	0.0246	0.6621	0.0247	0.6515	0.0414
13	342322	0.7000	0.6615	0.0249	0.6616	0.0251	0.6518	0.0378
14	412152	0.5010	0.6481	0.0258	0.6483	0.0259	0.6413	0.0389
15	423333	0.5725	0.6678	0.0224	0.6676	0.0222	0.6630	0.0359
16	431443	0.6133	0.6323	0.0248	0.6323	0.0250	0.6123	0.0379
17	442343	0.6500	0.5800	0.0258	0.5803	0.0256	0.5835	0.0388
18	513531	0.5992	0.7132	0.0230	0.7129	0.0231	0.6760	0.0378
19	531635	0.4386	0.5280	0.0231	0.5275	0.0229	0.5249	0.0358
20	535422	0.4771	0.6108	0.0248	0.6107	0.0247	0.6031	0.0368
21	542345	0.6446	0.5484	0.0246	0.5489	0.0243	0.5453	0.0367
22	545122	0.5517	0.6461	0.0227	0.6463	0.0225	0.6417	0.0367
23	614434	0.6523	0.6212	0.0277	0.6208	0.0276	0.6252	0.0387
24	625141	0.7030	0.5254	0.0242	0.5258	0.0243	0.5481	0.0384
25	635255	0.4560	0.3921	0.0218	0.3922	0.0219	0.4328	0.0385

**Table 2:** Impact of parameter uncertainty in the value set on estimates of a population’s mean health utility.

		PSA			Bayesian	
	Estimate	Standard Error (SE)	Variance	Estimate	SE	Variance
Mean health utility Usual estimate of uncertainty Uncertainty due to the value set	0.576	0.001689017	0.000002853	0.589	0.01284	0.000164866
		0.000353456	0.000000125		0.001778340	0.000003162
		0.001651620	0.000002728		0.012716254	0.000161703
Total			0.000002853			0.000164866

## Data Availability

Publicly available datasets have been used for this study.

## Supplementary Materials

The following are available online at https://www.mdpi.com/1660-4601/17/11/3949/s1, Table S1: Inference for the 249 health States.

## Abbreviations

HRQoL: Health-related quality of life; QALYs: Quality adjusted life years; EQ-5D: The EuroQol; SF-6D; Short form 6 dimensions health survey; SF-36: short form 36; HUI: Health Utilities Index; QWB: Quality of well-being; AQoL: Assessment of Quality of Life; SG: Standard gamble; TTO: Time trade-off; VAS: visual analogue scale; MVH: Measurement and Valuation of Health; PSA: probabilistic sensitivity analysis; NHS: National Health Service; NICE: National Institute for Health and Care Excellence; CEA: cost-effectiveness analysis; MCMC: Markov Chain Monte Carlo; GLS: Generalized least square; RMSE: Root mean square error; SD: Standard deviation; N(.,.): Normal distribution; UK: United Kingdom; HK: Hong Kong.
