# Supplementary material for: The Importance of Accounting for Parameter Uncertainty in SF-6D Value Sets and Its Impact on Studies that Use the SF-6D to Measure Health Utility
Source: Int J Environ Res Public Health. 2020 Jun 2;17(11):3949. doi: 10.3390/ijerph17113949 (PMC7311987; doi:10.3390/ijerph17113949)
Supplement: Supplementary file 1 [file ijerph-17-03949-s001.pdf]

Supplementary material.

Table S1. Inference for the 249 health States.

		Brazier model [9]		PSA		Bayesian	
HS	Observed	Predicted	SD	Predicted	SD	Predicted	SD
111111	1.0000	1.0000	0.0000	1.0000	0.0000	1.0000	0.0000
111112	0.8957	0.9692	0.0139	0.9692	0.0139	0.9767	0.0343
111212	0.7133	0.9320	0.0197	0.9321	0.0197	0.9241	0.0386
111215	0.9008	0.8067	0.0242	0.8068	0.0242	0.7944	0.0375
111222	0.8914	0.8962	0.0223	0.8962	0.0224	0.9099	0.0385
111453	0.4962	0.7431	0.0249	0.7428	0.0251	0.7043	0.0392
111621	0.6200	0.7141	0.0239	0.7138	0.0242	0.7096	0.0377
112111	0.8211	0.9465	0.0135	0.9465	0.0133	0.9554	0.0334
112221	0.8250	0.8735	0.0224	0.8735	0.0222	0.8689	0.0371
112521	0.7140	0.7468	0.0237	0.7467	0.0238	0.7402	0.0378
112543	0.6070	0.6648	0.0252	0.6648	0.0253	0.6386	0.0393
113411	0.5967	0.9283	0.0207	0.9282	0.0207	0.8928	0.0379
114212	0.6238	0.8053	0.0248	0.8053	0.0249	0.7888	0.0395
114244	0.5681	0.7150	0.0242	0.7150	0.0241	0.6982	0.0361
115653	0.5813	0.5206	0.0242	0.5201	0.0243	0.5347	0.0394
121111	0.9278	0.9495	0.0122	0.9496	0.0122	0.9687	0.0334
121112	0.6775	0.9187	0.0176	0.9188	0.0176	0.8967	0.0360
121122	0.8350	0.8828	0.0210	0.8828	0.0210	0.8784	0.0369
121212	0.7829	0.8815	0.0227	0.8817	0.0227	0.8521	0.0395
122112	0.7989	0.8651	0.0209	0.8653	0.0209	0.8585	0.0368
122211	0.6927	0.8588	0.0227	0.8590	0.0224	0.8384	0.0377
122233	0.8271	0.7951	0.0276	0.7949	0.0272	0.7795	0.0383
122425	0.6570	0.6641	0.0273	0.6641	0.0271	0.6563	0.0396
122622	0.7267	0.5792	0.0259	0.5791	0.0262	0.6003	0.0396
122653	0.4817	0.5122	0.0248	0.5119	0.0249	0.5144	0.0372
124114	0.7300	0.8013	0.0221	0.8012	0.0222	0.7883	0.0383
124314	0.7050	0.7672	0.0252	0.7671	0.0251	0.7669	0.0387
131542	0.4235	0.6476	0.0245	0.6477	0.0245	0.6024	0.0380
132425	0.6573	0.6566	0.0263	0.6564	0.0263	0.6421	0.0387
132524	0.5800	0.6672	0.0264	0.6671	0.0266	0.6415	0.0410
133132	0.5691	0.7503	0.0247	0.7500	0.0246	0.7212	0.0391
133511	0.7220	0.7462	0.0227	0.7461	0.0227	0.7441	0.0353
134322	0.5707	0.7144	0.0247	0.7142	0.0248	0.6867	0.0368
134352	0.6609	0.6347	0.0254	0.6345	0.0253	0.6374	0.0375
135332	0.7250	0.6523	0.0274	0.6521	0.0273	0.6507	0.0390
141653	0.3875	0.5537	0.0234	0.5533	0.0234	0.5585	0.0368

142154	0.5110	0.6635	0.0247	0.6636	0.0245	0.6657	0.0382
142631	0.4688	0.5883	0.0251	0.5880	0.0250	0.5872	0.0393
143611	0.6500	0.6555	0.0224	0.6554	0.0224	0.6646	0.0360
144113	0.4863	0.7926	0.0229	0.7924	0.0229	0.7618	0.0394
144144	0.6842	0.6896	0.0221	0.6897	0.0219	0.6718	0.0364
144241	0.7656	0.6739	0.0257	0.6741	0.0255	0.6567	0.0398
144341	0.7267	0.6769	0.0228	0.6771	0.0226	0.6789	0.0320
145133	0.5736	0.6946	0.0253	0.6943	0.0250	0.6938	0.0385
145353	0.5363	0.5905	0.0245	0.5905	0.0242	0.5911	0.0394
211111	0.7780	0.9500	0.0155	0.9500	0.0154	0.9359	0.0341
211211	0.7944	0.9129	0.0197	0.9129	0.0197	0.8927	0.0364
211212	0.8900	0.8820	0.0226	0.8821	0.0227	0.8939	0.0392
211221	0.7188	0.8770	0.0227	0.8769	0.0228	0.8595	0.0384
212442	0.5629	0.6428	0.0266	0.6429	0.0266	0.6184	0.0386
212453	0.6483	0.6396	0.0259	0.6393	0.0261	0.6406	0.0381
213114	0.7775	0.8132	0.0240	0.8132	0.0237	0.7923	0.0372
213323	0.7433	0.8300	0.0293	0.8297	0.0294	0.8224	0.0402
213345	0.4270	0.6282	0.0271	0.6283	0.0268	0.6105	0.0400
214411	0.7442	0.7835	0.0251	0.7832	0.0248	0.7648	0.0379
214535	0.6258	0.6246	0.0259	0.6240	0.0258	0.6090	0.0383
215154	0.4769	0.6339	0.0258	0.6337	0.0256	0.6368	0.0379
221211	0.7680	0.8623	0.0222	0.8624	0.0222	0.8587	0.0365
221212	0.7233	0.8315	0.0242	0.8317	0.0242	0.8355	0.0362
221432	0.8056	0.7833	0.0259	0.7830	0.0259	0.7991	0.0395
221535	0.6960	0.6174	0.0255	0.6171	0.0254	0.6247	0.0387
222113	0.7319	0.8278	0.0242	0.8278	0.0244	0.8051	0.0364
222121	0.6455	0.8101	0.0245	0.8101	0.0245	0.7862	0.0390
222122	0.6769	0.7793	0.0248	0.7793	0.0250	0.7707	0.0363
222212	0.6963	0.7780	0.0261	0.7782	0.0261	0.7646	0.0402
223451	0.6743	0.6288	0.0275	0.6287	0.0275	0.6547	0.0425
223511	0.7033	0.7038	0.0241	0.7038	0.0237	0.6778	0.0368
224112	0.7340	0.7419	0.0244	0.7419	0.0242	0.7471	0.0382
224223	0.8021	0.6816	0.0260	0.6813	0.0261	0.6972	0.0383
224612	0.5400	0.5753	0.0263	0.5750	0.0262	0.5715	0.0392
232111	0.7589	0.7550	0.0251	0.7550	0.0247	0.7283	0.0395
233551	0.7708	0.5806	0.0257	0.5804	0.0255	0.5934	0.0380
234233	0.5185	0.6644	0.0262	0.6637	0.0261	0.6504	0.0378
234551	0.4011	0.5692	0.0253	0.5689	0.0252	0.5636	0.0388
235224	0.4682	0.6183	0.0281	0.6182	0.0279	0.6009	0.0404
241531	0.7529	0.6780	0.0245	0.6778	0.0243	0.6777	0.0358
241545	0.5965	0.5512	0.0244	0.5514	0.0243	0.5508	0.0321
241635	0.5440	0.5191	0.0232	0.5187	0.0232	0.5566	0.0372
243432	0.7753	0.6560	0.0258	0.6557	0.0257	0.6672	0.0376
243634	0.5118	0.5385	0.0254	0.5380	0.0254	0.5654	0.0389
244313	0.6343	0.7085	0.0265	0.7083	0.0263	0.7023	0.0406

311222	0.8429	0.8579	0.0238	0.8579	0.0238	0.8475	0.0389
311233	0.7127	0.8609	0.0247	0.8605	0.0245	0.8352	0.0388
311655	0.5031	0.5235	0.0218	0.5231	0.0221	0.5168	0.0364
312255	0.6458	0.5994	0.0232	0.5994	0.0234	0.6105	0.0314
312332	0.7775	0.7978	0.0276	0.7976	0.0275	0.7646	0.0407
312455	0.6967	0.5968	0.0227	0.5966	0.0230	0.6167	0.0370
312552	0.5300	0.5980	0.0253	0.5980	0.0254	0.5915	0.0368
313532	0.6146	0.6897	0.0247	0.6894	0.0248	0.6746	0.0375
314631	0.5600	0.6229	0.0263	0.6222	0.0265	0.6214	0.0384
315515	0.5587	0.6295	0.0259	0.6294	0.0258	0.6342	0.0370
321122	0.7571	0.8445	0.0216	0.8446	0.0215	0.8306	0.0344
321144	0.6164	0.7067	0.0214	0.7069	0.0215	0.6798	0.0310
321221	0.8400	0.8382	0.0239	0.8383	0.0238	0.8134	0.0390
321335	0.7706	0.6755	0.0240	0.6753	0.0239	0.6792	0.0359
321455	0.5950	0.5998	0.0235	0.5997	0.0237	0.5971	0.0377
322134	0.7150	0.7073	0.0248	0.7071	0.0249	0.6847	0.0388
322635	0.4867	0.4894	0.0240	0.4890	0.0241	0.4998	0.0372
322644	0.5738	0.4865	0.0247	0.4866	0.0251	0.4909	0.0373
323135	0.6046	0.6777	0.0228	0.6774	0.0229	0.6833	0.0362
323153	0.5656	0.6623	0.0241	0.6621	0.0240	0.6427	0.0357
323333	0.6757	0.7816	0.0256	0.7812	0.0255	0.7801	0.0404
323431	0.7978	0.7940	0.0263	0.7937	0.0262	0.8054	0.0398
323433	0.5647	0.7759	0.0266	0.7754	0.0265	0.7604	0.0383
323443	0.4925	0.6383	0.0244	0.6383	0.0247	0.6188	0.0378
323632	0.6442	0.5530	0.0241	0.5525	0.0242	0.5716	0.0378
323644	0.3970	0.5082	0.0235	0.5081	0.0238	0.5040	0.0374
323645	0.4473	0.4569	0.0236	0.4569	0.0237	0.4593	0.0359
324125	0.7069	0.6759	0.0240	0.6758	0.0242	0.6713	0.0358
325455	0.4686	0.5041	0.0240	0.5040	0.0241	0.5230	0.0367
331244	0.7450	0.6620	0.0246	0.6621	0.0247	0.6515	0.0414
332113	0.7593	0.7486	0.0249	0.7485	0.0249	0.7300	0.0368
332145	0.6973	0.5943	0.0230	0.5945	0.0230	0.5997	0.0372
332411	0.7700	0.7269	0.0274	0.7269	0.0276	0.7028	0.0392
333154	0.6365	0.6514	0.0226	0.6512	0.0226	0.6592	0.0346
333225	0.6358	0.6426	0.0253	0.6425	0.0256	0.6335	0.0379
333333	0.6389	0.6906	0.0225	0.6901	0.0225	0.6909	0.0373
333433	0.5357	0.6849	0.0236	0.6843	0.0237	0.6740	0.0386
334254	0.5838	0.6028	0.0255	0.6025	0.0257	0.5876	0.0376
341123	0.7570	0.7618	0.0242	0.7617	0.0245	0.7475	0.0383
342322	0.7000	0.6615	0.0249	0.6616	0.0251	0.6518	0.0378
342353	0.6611	0.5945	0.0254	0.5944	0.0256	0.6090	0.0394
343214	0.6840	0.7253	0.0269	0.7254	0.0271	0.7135	0.0393
343312	0.7282	0.7190	0.0258	0.7192	0.0260	0.6980	0.0389
343325	0.7718	0.6412	0.0244	0.6412	0.0246	0.6569	0.0377
344145	0.5145	0.6001	0.0235	0.6002	0.0238	0.5945	0.0373

344344	0.7111	0.6172	0.0250	0.6173	0.0253	0.6079	0.0388
345122	0.6354	0.6534	0.0241	0.6535	0.0241	0.6583	0.0367
345623	0.5862	0.4994	0.0247	0.4991	0.0248	0.5314	0.0380
411245	0.7043	0.6384	0.0248	0.6388	0.0250	0.6383	0.0367
412152	0.5010	0.6481	0.0258	0.6483	0.0259	0.6413	0.0389
413144	0.6785	0.6949	0.0220	0.6952	0.0219	0.6954	0.0355
413333	0.7786	0.7183	0.0225	0.7180	0.0226	0.7280	0.0365
413414	0.6770	0.7548	0.0249	0.7549	0.0249	0.7232	0.0394
413511	0.6186	0.7357	0.0220	0.7358	0.0220	0.7144	0.0358
414511	0.8443	0.7242	0.0232	0.7242	0.0231	0.7147	0.0385
414522	0.5409	0.6575	0.0260	0.6575	0.0260	0.6416	0.0385
415424	0.8467	0.6551	0.0271	0.6551	0.0269	0.6785	0.0405
421314	0.7125	0.7419	0.0265	0.7421	0.0263	0.7023	0.0398
422655	0.3900	0.3890	0.0239	0.3890	0.0240	0.4056	0.0366
423333	0.5725	0.6678	0.0224	0.6676	0.0222	0.6630	0.0359
423343	0.4414	0.6136	0.0237	0.6139	0.0236	0.5897	0.0368
423433	0.5750	0.6621	0.0229	0.6618	0.0228	0.6570	0.0373
424421	0.5925	0.6785	0.0252	0.6784	0.0251	0.6602	0.0368
424554	0.4946	0.5367	0.0254	0.5366	0.0254	0.5420	0.0332
424643	0.6275	0.4697	0.0248	0.4696	0.0249	0.4790	0.0393
425133	0.7700	0.6381	0.0264	0.6379	0.0263	0.6343	0.0417
425521	0.5090	0.5854	0.0263	0.5856	0.0261	0.5792	0.0393
431144	0.6786	0.6687	0.0217	0.6690	0.0216	0.6488	0.0383
431435	0.6440	0.6318	0.0243	0.6316	0.0242	0.6218	0.0362
431443	0.6133	0.6323	0.0248	0.6323	0.0250	0.6123	0.0379
431623	0.4507	0.5692	0.0253	0.5689	0.0253	0.5581	0.0363
432255	0.4818	0.5109	0.0253	0.5111	0.0252	0.5084	0.0384
432623	0.5458	0.5157	0.0256	0.5154	0.0256	0.5190	0.0375
433142	0.6550	0.6275	0.0242	0.6278	0.0239	0.6366	0.0365
433333	0.7067	0.6602	0.0221	0.6599	0.0219	0.6597	0.0357
433433	0.7222	0.6545	0.0230	0.6541	0.0229	0.6414	0.0378
433541	0.6500	0.5779	0.0237	0.5781	0.0238	0.5698	0.0375
434654	0.3863	0.4429	0.0246	0.4425	0.0245	0.4429	0.0363
441132	0.5873	0.7090	0.0252	0.7091	0.0249	0.6765	0.0397
442343	0.6500	0.5800	0.0258	0.5803	0.0256	0.5835	0.0388
443144	0.5042	0.6324	0.0232	0.6328	0.0230	0.6347	0.0365
443215	0.6725	0.6436	0.0265	0.6440	0.0266	0.6426	0.0386
443222	0.6785	0.6497	0.0262	0.6500	0.0263	0.6697	0.0385
443335	0.5062	0.6012	0.0239	0.6011	0.0236	0.5869	0.0376
445321	0.5962	0.6197	0.0255	0.6200	0.0253	0.6212	0.0378
511114	0.6038	0.8496	0.0215	0.8496	0.0214	0.7993	0.0382
512242	0.7045	0.6499	0.0267	0.6503	0.0266	0.6366	0.0394
512551	0.4709	0.6215	0.0236	0.6215	0.0236	0.6069	0.0374
513354	0.7014	0.6680	0.0247	0.6680	0.0249	0.6659	0.0363
513531	0.5992	0.7132	0.0230	0.7129	0.0231	0.6760	0.0378

515332	0.6892	0.6649	0.0262	0.6647	0.0263	0.6682	0.0379
521424	0.5138	0.7234	0.0244	0.7233	0.0243	0.6810	0.0376
522321	0.6755	0.6970	0.0250	0.6972	0.0248	0.6762	0.0385
523554	0.5457	0.5712	0.0252	0.5712	0.0253	0.5638	0.0399
523634	0.6067	0.5550	0.0244	0.5546	0.0246	0.5524	0.0374
524442	0.5407	0.6069	0.0250	0.6070	0.0249	0.6155	0.0374
524644	0.4947	0.4894	0.0225	0.4893	0.0226	0.4740	0.0350
525112	0.5017	0.6940	0.0253	0.6942	0.0251	0.6790	0.0406
525311	0.7792	0.6907	0.0263	0.6909	0.0260	0.6971	0.0382
531635	0.4386	0.5280	0.0231	0.5275	0.0229	0.5249	0.0358
532124	0.7892	0.7021	0.0226	0.7021	0.0225	0.6900	0.0361
532455	0.5613	0.5314	0.0238	0.5313	0.0239	0.5441	0.0361
532554	0.6922	0.5420	0.0232	0.5419	0.0231	0.5419	0.0379
533331	0.8008	0.7014	0.0234	0.7012	0.0234	0.6902	0.0368
534133	0.6018	0.7059	0.0242	0.7054	0.0241	0.6756	0.0387
534544	0.5917	0.5681	0.0216	0.5680	0.0217	0.5514	0.0392
534555	0.3488	0.5009	0.0231	0.5007	0.0232	0.4940	0.0385
534625	0.4417	0.4944	0.0246	0.4939	0.0248	0.4874	0.0373
534644	0.3500	0.4819	0.0228	0.4816	0.0228	0.4741	0.0367
535422	0.4771	0.6108	0.0248	0.6107	0.0247	0.6031	0.0368
535544	0.5100	0.5157	0.0235	0.5158	0.0235	0.5213	0.0383
535545	0.3600	0.4644	0.0239	0.4645	0.0239	0.4556	0.0372
535554	0.5222	0.4998	0.0227	0.4997	0.0226	0.5044	0.0378
535555	0.4800	0.4485	0.0220	0.4484	0.0219	0.4575	0.0371
535645	0.1000	0.3782	0.0241	0.3781	0.0240	0.3771	0.0402
541432	0.6958	0.6923	0.0245	0.6922	0.0242	0.6989	0.0375
541531	0.6447	0.6825	0.0231	0.6824	0.0228	0.6546	0.0359
541622	0.5038	0.5752	0.0226	0.5751	0.0225	0.5767	0.0359
542325	0.5582	0.6122	0.0244	0.6124	0.0244	0.5951	0.0384
542345	0.6446	0.5484	0.0246	0.5489	0.0243	0.5453	0.0367
542524	0.5008	0.6172	0.0249	0.6173	0.0249	0.6081	0.0376
543344	0.5056	0.6213	0.0235	0.6217	0.0233	0.6155	0.0375
543624	0.4843	0.5526	0.0235	0.5524	0.0237	0.5622	0.0385
544223	0.7515	0.6740	0.0259	0.6739	0.0262	0.6669	0.0380
544352	0.4280	0.5847	0.0250	0.5847	0.0252	0.5581	0.0394
544555	0.4500	0.4965	0.0235	0.4964	0.0237	0.4985	0.0354
544633	0.6779	0.5349	0.0237	0.5342	0.0236	0.5450	0.0362
544644	0.4083	0.4774	0.0219	0.4773	0.0219	0.4730	0.0386
544654	0.3529	0.4615	0.0219	0.4612	0.0221	0.4762	0.0384
545122	0.5517	0.6461	0.0227	0.6463	0.0225	0.6417	0.0367
545422	0.6044	0.6063	0.0240	0.6064	0.0238	0.6048	0.0382
545523	0.5800	0.5783	0.0248	0.5782	0.0248	0.5941	0.0389
545622	0.6975	0.4795	0.0215	0.4794	0.0215	0.5035	0.0364
545644	0.2500	0.4250	0.0220	0.4251	0.0219	0.4241	0.0378
545654	0.3988	0.4091	0.0201	0.4090	0.0201	0.4467	0.0382

545655	0.2263	0.3579	0.0179	0.3577	0.0180	0.3765	0.0373
612442	0.2863	0.5475	0.0270	0.5478	0.0271	0.5583	0.0406
613143	0.6418	0.6216	0.0256	0.6217	0.0256	0.6442	0.0388
614321	0.5008	0.6580	0.0253	0.6580	0.0253	0.6383	0.0385
614434	0.6523	0.6212	0.0277	0.6208	0.0276	0.6252	0.0387
615144	0.6488	0.5545	0.0228	0.5548	0.0231	0.5696	0.0382
621221	0.4946	0.6478	0.0267	0.6480	0.0268	0.6320	0.0385
621451	0.6633	0.5655	0.0259	0.5655	0.0259	0.5961	0.0417
622513	0.5669	0.5687	0.0281	0.5688	0.0281	0.5679	0.0388
623133	0.6138	0.6252	0.0252	0.6250	0.0251	0.6448	0.0390
624115	0.6207	0.6048	0.0236	0.6049	0.0236	0.6140	0.0359
624142	0.5388	0.5470	0.0237	0.5473	0.0236	0.5342	0.0355
624331	0.5400	0.5978	0.0249	0.5977	0.0246	0.5941	0.0372
624343	0.4836	0.5256	0.0245	0.5257	0.0244	0.5294	0.0365
625141	0.7030	0.5254	0.0242	0.5258	0.0243	0.5481	0.0384
625213	0.7408	0.5698	0.0271	0.5700	0.0270	0.6129	0.0406
631231	0.5700	0.6305	0.0265	0.6304	0.0266	0.6028	0.0396
631354	0.4923	0.5421	0.0257	0.5421	0.0255	0.5421	0.0374
631355	0.6573	0.4909	0.0237	0.4909	0.0235	0.5298	0.0373
632121	0.6092	0.6238	0.0248	0.6239	0.0250	0.6160	0.0375
633122	0.4663	0.6147	0.0260	0.6147	0.0261	0.6116	0.0398
634124	0.6920	0.6126	0.0240	0.6124	0.0242	0.6246	0.0379
634545	0.3038	0.4171	0.0242	0.4171	0.0244	0.4210	0.0371
635244	0.5000	0.4592	0.0256	0.4595	0.0258	0.4801	0.0392
635255	0.4560	0.3921	0.0218	0.3922	0.0219	0.4328	0.0385
635544	0.4250	0.4160	0.0251	0.4161	0.0255	0.4290	0.0388
635554	0.4013	0.4001	0.0235	0.4000	0.0236	0.4382	0.0399
641424	0.6942	0.6116	0.0271	0.6117	0.0272	0.6332	0.0390
641622	0.4775	0.4755	0.0238	0.4754	0.0238	0.4940	0.0376
642612	0.4844	0.4578	0.0261	0.4579	0.0259	0.4893	0.0372
644545	0.4433	0.4126	0.0243	0.4128	0.0243	0.4507	0.0402
645555	0.4367	0.3444	0.0194	0.3445	0.0194	0.3986	0.0411
645655	0.2130	0.2582	0.0129	0.2581	0.0130	0.2449	0.0136
